# Preoperative predictors of implant size in patients undergoing total knee arthroplasty: a retrospective cohort study

**DOI:** 10.1186/s12891-023-06785-0

**Published:** 2023-08-15

**Authors:** Mohsen Ostovar, Mahmoud Jabalameli, Mohammad Reza Bahaeddini, Abolfazl Bagherifard, Mansour Bahardoust, Alireza Askari

**Affiliations:** 1 Bone and Joint Reconstruction Research Center, Shafa Orthopedic Hospital, Iran University of Medical Sciences, Baharestan Square, 1157637131, Tehran, Iran; 2 Department of Epidemiology, School of Public Health, Shahid Beheshti University of Medical Sciences, Tehran, Iran

**Keywords:** Total knee arthroplasty, Tibial component, Femoral component, Implant size, Demographics, Ankle volume

## Abstract

**Background:**

Traditionally, the size of total knee arthroplasty (TKA) components is predicted by preoperative radiographic templating, which is of limited accuracy. This study aimed to evaluate the role of demographic data and ankle volume in predicting implant size in TKA candidates.

**Methods:**

In a retrospective study, 415 patients who underwent TKA at a single institution were included. The mean age of the patients was 67.5 ± 7.1 years. The mean BMI of the patients was 31.1 ± 4.7 kg/m^2^. TKA implants were Zimmer Biomet NexGen LPS-Flex Knee in all cases. The demographic data included age, sex, height, weight, BMI, ethnicity, and ankle volume. Ankle volume was assessed with the figure-of-eight method. Multivariate linear regression analysis was used for predicting factors of implant size.

**Results:**

Multivariate linear regression analysis showed that the Sex (β:1.41, P < 0.001), height (β:0.058, P < 0.001), ankle volume (β:0.11, P < 0.001), and Age (β:0.017, P = 0.004) were significant predictors of tibial component size. Sex (β:0.89, P < 0.001), height (β:0.035, P < 0.001), and ankle volume(β:0.091, P < 0.001) were significant predictors of femoral component size in the multivariate analysis.

**Conclusion:**

Demographic data, adjunct with the ankle volume, could provide a promising model for preoperative prediction of the size of tibial and femoral components in TKA candidates.

## Introduction

The appropriate size of the femoral and tibial components is essential for achieving an acceptable alignment and correct lower limb balance in patients undergoing total knee arthroplasty (TKA) [1, 2]. In posterior reference systems, smaller size results in notching in the anterior cortex of the femur, while in anterior reference systems, a smaller size results in flexion gap enlargement and knee instability [3, 4]. Therefore, developing reliable strategies to preoperatively predict the appropriate size of TKA components, specifically in patients with sizes outside the normal range, is particularly important.

Preoperative radiographic templating is used to estimate the size of the femoral and tibial TKA components. But the accuracy of this method in predicting the size of tibia and femur components, unlike total hip arthroplasty(THA), is less and depends on various factors, including the position and accuracy of the X-ray marker. So in the study of Ooka et al., radiographic templating to predict prosthesis size was only correct in 28.2% of cases using anteroposterior radiography and 35.9% of cases using lateral radiography [5]. In addition, preoperative radiographic templating is time-consuming, difficult, and costly [6]. However, the X-ray pattern can still help predict the implant size with a slight deviation, especially in THA [7].

Recent studies have shown that patients’ demographic characteristics have a higher potential in predicting the size of TKA components than preoperative radiographic templating [8–11]. Blevins et al. developed a Bayesian model to predict the TKA component size according to the demographic characteristics of the patients. Their model showed high reliability in preoperative prediction of the size of TKA implants, with an accuracy of more than 90% [9].

Since demographic characteristics are influenced by factors such as ethnicity and race and the ankle’s size is different in different races [12, 13],the association of TKA components with these data should be determined population-specifically [8].

However, studies have yet to be conducted in Iran to evaluate the relationship between demographic findings and the size of femoral and tibial TKA components. In this study, we aimed to evaluate the association of demographic characteristics with the size of TKA components in different Iranian ethnic groups. We also evaluated the association of ankle volume with the size of TKA components that were not included in any earlier investigation.

## Methods

The ethics board approved this study of our institute under the code IR.IUMS.REC.1401.134. In a prospective study, the medical profiles of the patients who underwent TKA at our hospital between 2019 and 2022 were retrospectively reviewed. The inclusion criteria were primary TKA and normal growth. Patients with bone deformity, unicompartmental knee arthroplasty, conversion from a unicompartmental arthroplasty, Patients with known pathological ankle disorders, patients with obvious ankle osteoarthritis on radiographs, congenital joint disorders, previous history of trauma with permanent joint swelling, patients with systemic diseases such as cardiovascular and kidney diseases, which were associated with persistent and irreversible ankle swelling, and those who underwent TKA for fracture treatment were excluded from the study. The eligible patients were called and asked to attend an evaluation session to measure ankle volume. Of 468 eligible patients, 415 attended the final evaluation session and were included in the analysis. All patients were informed of the study protocol and provided written informed consent informed prior to participation in the study.

**Fig. 1:**
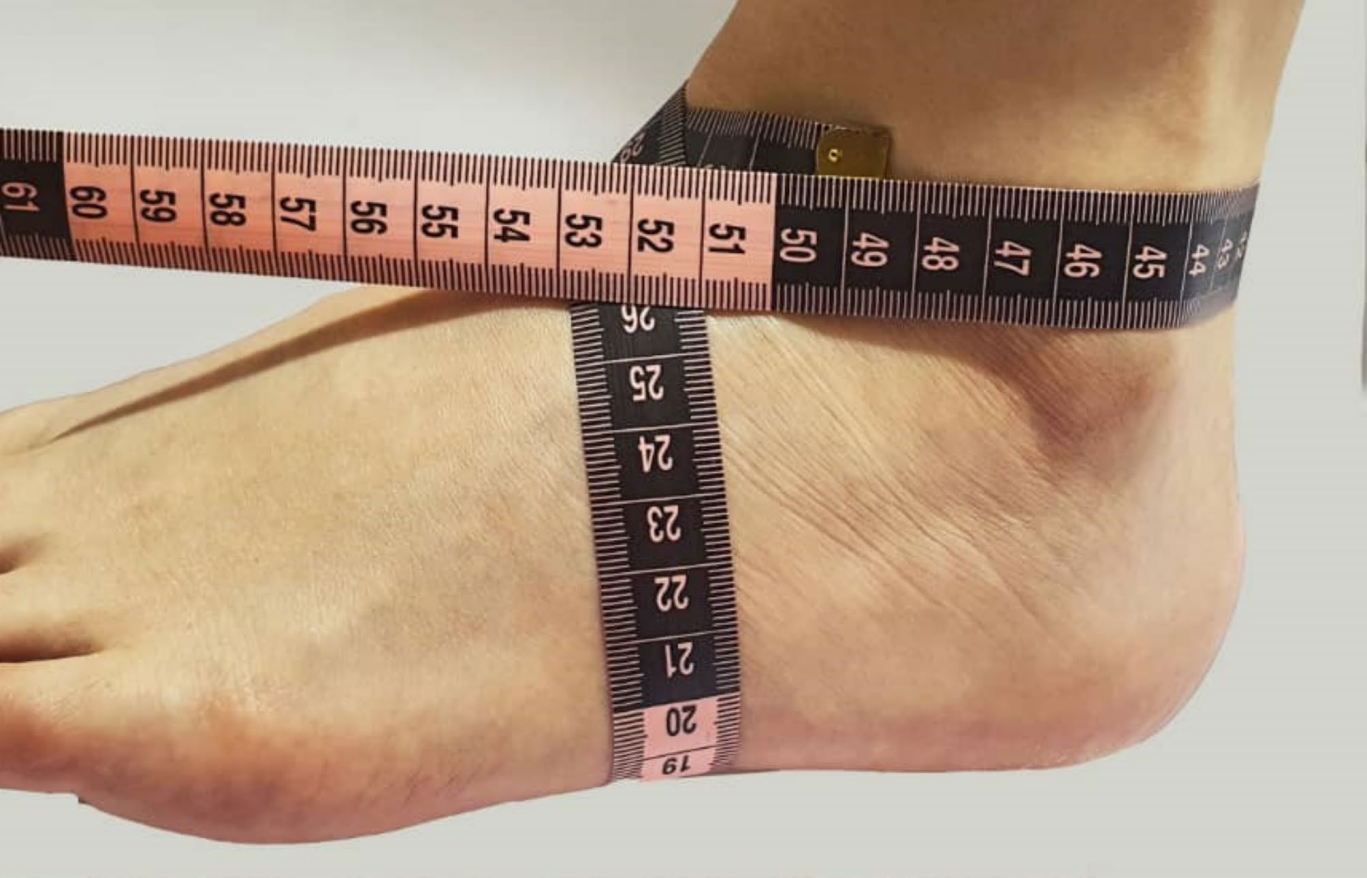
Evaluation of ankle volume using the figure-of-eight technique

Demographic characteristics of the patients, including age, sex, BMI, laterality, and ethnicity, were extracted from the patient’s medical profiles. The ankle volume was evaluated in the evaluation session using the figure-of-eight technique [14]. To this aim, the patient was placed in a long sitting position while the ankle was kept in a neutral position. The zero point of the measuring tape was located midway over the anterior ankle joint prominence, between the prominence of the anterior tibialis tendon and lateral malleolus. Then the measuring tape was directed over the navicular tuberosity through the center of the medial longitudinal arch of the ankle and gently touched the sole toward the lateral malleolus, Achilles tendon, and medial malleolus to finish at the zero point (Fig. 1).

The size of the femoral and tibial TKA components was extracted from the patients’ profiles. The Zimmer Biomet NexGen LPS-Flex Knee implant (Zimmer Biomet, Warsaw, Indiana, USA) was used in all cases. The anteroposterior (AP) dimension of the femoral component for Zimmer implants was categorized into A to G in alphabetic order. The tibial component’s mediolateral (ML) dimension was categorized into 1 to 10. (Table 1)

**Table 1:** Zimmer Biomet NexGen LPS-Flex feoral and tibial complonent dimentions

Femoral size	A	B	C	D	E	F	G			
AP diameter	46.5	50.3	45.6	58.6	62.5	66.5	71.6			
ML diameter	54	58	60	64	68	72	76.5			
Tibial size	**1**	**2**	**3**	**4**	**5**	**6**	**7**	**8**	**9**	**10**
AP diameter	40	41	42	46	46	50	51	54	43	57
ML diameter	58	62	66	66	74	74	82	82	89	89

AP: antroposterior; ML: mediolateral

The association of the patients’ characteristics with the TKA components was analyzed separately for two types of prostheses. Two senior fellowship-trained knee surgeons performed the surgeries at a single institution.

### Statistical analysis

SPSS version 16 software (SPSS Inc., Chicago, Ill., USA) was used to analyze the data. Descriptive information was demonstrated by mean and standard deviations (for quantitative data) or numbers and percentages (for qualitative data). A univariate analysis first evaluated the associations between the patient’s characteristics and TKA component size. Variables with p < 0.15 in univariate analysis entered the multivariate analysis model of linear regression with the backward model. P values less than 0.05 were considered significant.

## Results

The study population included 123 males and 292 females with a mean age of 67.5 ± 7.1 years. The mean BMI of the patients was 31.1 ± 4.7 kg/m^2^. The baseline characteristics of the patients and implants are demonstrated in Table 2. The distribution of the tibial and femoral size is shown in Table 3.

**Table 2:** baseline characteristics of the TKA candidates

Variable	Mean ± SD or number (%) (n = 415)
Age (year)	67.5 ± 7.1
Sex	
• Male • Female	123 (29.6) 292 (70.4)
Laterality	
• Right • Left	206 (49.8) 208 (50.2)
Weight (kg)	77.6 ± 114.3
Height (cm)	157.6 ± 9.4
BMI (Kg/m^2^)	31.1 ± 4.7
Ethnicity	
• Persian • Azerbaijanians • Lur • Kurd	223 (53.7) 139 (33.5) 37 (8.9) 16 (3.9)

Data are demonstrated as mean ± SD or number (%)

**Table 3:** Distribution of the tibial and femoral component size

Component	Size	Frequency(%)
Tibial component	1	86 (20.7)
	2	107 (25.8)
	3	109 (26.3)
	4	43 (10.4)
	5	53 (12.8)
	6	17 (4.1)
Femoral component	A	2 (0.5)
	B	16 (3.9)
	C	100 (24.1)
	D	117 (28.2)
	E	91 (21.9)
	F	86 (20.7)
	G	3 (0.7)

Data are demonstrated as numbers (%)

In univariate regression analysis, the size of the tibial component was significantly associated with age (P < 0.001), sex (P < 0.001), height (P < 0.001), weight (P < 0.001), and ankle volume (P < 0.001). The femoral component size showed the same associations with the patient’s characteristics in univariate analysis (Table 4).

**Table 4:** Univariate analysis showing the predictive value of patient’s characteristics for tibial and femoral components size

Variable	Tibial component size				Femoral component size			
	R2	Beta	SE of beta	P-value	R2	Beta	SD	P-value
Age	0.041	0.041	0.010	< 0.001	0.029	0.029	0.008	< 0.001
Height	0.483	0.106	0.005	< 0.001	0.421	0.084	0.005	< 0.001
Weight	0.133	0.036	0.005	< 0.001	0.119	0.029	0.004	< 0.001
BMI	0.004	-0.020	0.015	0.178	0.001	-0.009	0.013	0.460
Ankle volume	0.344	0.237	0.016	< 0.001	0.334	0.197	0.014	< 0.001
Sex	0.550	2.316	0.103	< 0.001	0.423	1.713	0.098	< 0.001
Ethnicity	0.001	-0.020	0.039	0.608	0.002	-0.028	0.033	0.390
Side	0.001	0.094	0.141	0.506	0.001	-0.003	0.118	0.979

P < 0.05 is considered significant., SD: Standar devintion

Sex, ethnicity, weight, height, and ankle volume were included in a linear regression analysis. In this model, sex, weight, height, and ankle volume were still significantly associated with the size of the tibial components. The model generated using these variables correctly predicted the size of the tibial component in 77% of cases (Table 4).

Multivariate linear regression analysis shows that the Sex (β:1.41, P < 0.001), height (β:0.058, P < 0.001), ankle volume (β:0.11, P < 0.001), and Age (β:0.017, P = 0.004) were significant predictors of tibial component size. Sex (β:0.89, P < 0.001), height (β:0.035, P < 0.001), and ankle volume(β:0.091, P < 0.001) were significant predictors of femoral component size in the multivariate analysis (Table 5).

**Table 5:** Multivariate regression analysis for the prediction of the tibial and femoral component size

Variable	Tibial component size (R2 = 0.678, Adj. R2 = 0.673)			Femoral component size (R2 = 0.669, Adj. R2 = 0.664)		
	β	95% CI	P value	β	95% CI	P value
Sex (male vs. female)	1.41	1.19,1.62	0.001	0.89	0.69,1.1	0.001
Height (CM)	0.058	0.047,0.069	0.001	0.035	0.033,0.036	0.001
Ankle volume (cm^3^)	0.11	0.07,0.16	0.001	0.095	0.089,0.15	0.001
Age (Per Year)	0.017	0.007, 0.028	0.004	0.0031	-0.011,0.018	0.51

*95% CI: 95 Confidence Interval

## Discussion

In this study, we evaluated the role of demographic characteristics and ankle volume of TKA candidates in the preoperative prediction of the size of femoral and tibial components. In multimodal regression analysis, the patient’s age, sex, height, and ankle volume were significantly associated with the size of the tibial component. Also, the patient’s sex, height, and ankle volume were significantly associated with the size of the femoral component. The model generated using these variables correctly predicted the size of the tibial and femoral components in 77% and 81.1% of cases, respectively.

Several recent studies have used the patients’ demographic data to predict the size of TKA components. Blevins et al. performed a retrospective study to determine the association of demographic characteristics, including height, weight, and sex, with implant size in TKA candidates. According to their analysis, the implant size showed a significant linear correlation with height, weight, and sex. Accordingly, they generated a model that could predict the size of the tibial and femoral components with an accuracy of more than 90% [9]. Similar to the study of Blevins et al., height and sex were significantly associated with the implant size in the present study.

Wallace et al. aimed to investigate the role of demographic characteristics, including age, sex, height, weight, and ethnicity, in predicting implant size in a consecutive series of 201 patients undergoing primary TKA. In multivariate analysis, all the demographic characteristics significantly predicted TKA implant size. The regression model correctly predicted the size of the femoral and tibial component in 43.7 and 43.7% of cases, respectively, while radiographic templating correctly predicted the size of the femoral and tibial component in 35.4 and 36.5% of cases, respectively. They concluded that the regression model generated by the demographic data more accurately predicts implanted component sizes compared to radiographic templating [8]. In the present study, ethnicity was not significantly associated with the size of TKA implants in univariate and multivariate analyses. This inconsistency resulted from a small number of patients in some ethnicity subgroups.

Marino et al. used patient demographic characteristics (height, weight, BMI, sex, age) to predict the size of TKA components preoperatively in 484 patients undergoing a primary TKA at a single institution. Height, weight, and sex predicted the implanted component size with an accuracy of 54.0% for the tibia and 51.1% for the femur. These results were highly correlated to the tibial and femoral component size predicted by Arthroplasty Size Predictor [15].

Sershon et al. aimed to determine whether the patient’s height, weight, and sex can accurately predict the size of TKA components. They included a consecutive series of 3491 primary TKA patients. The height, weight, and sex were significantly correlated with the size of the femoral and tibial components. Accordingly, the femoral and tibial sizes were correctly predicted within one size of the final implant in 71-92% and 81-97% of cases, respectively. When combined with preoperative templating data, the model correctly predicted the size of the femoral and tibial components within one size of the final implant in 85% and 90% of cases, respectively [16]. We did not evaluate the role of preoperative radiographic templating in the present study.

Since the Zimmer prosthesis is designed based on the anatomic and anthropometric characteristics of the American population, which considerably differs from that in Middle Eastern countries, such as Iran [17], the role of anthropometric data in predicting the size of TKA components has been investigated in some other studies and reported to be a promising predictor of implant size in the majority of the published articles [18, 19]. Some orthopedic centers, particularly in developing and underdeveloped countries, generally need different sizes of TKA components, and access to all components is not feasible. For this reason, preoperative predicting the size of the required prosthesis and checking the size availability is of considerable importance. Traditional radiographic templating used to be used for the preoperative prediction of TKA component sizes. However, such templating could be of more accuracy [5]. The development of modern digital X-ray devices with various magnifications has made radiographic templating even less reliable [20]. Therefore, formulas capable of preoperative predicting the sizes of TKA components, such as the formula presented in this study, could be of considerable importance.

Limited studies have investigated the relationship between ankle volume and implant size. The study by Trainor et al. revealed that shoe size is more accurate than body height in the prediction of implant size in patients undergoing TKA [21]. The same results were reported by Rehman et al. [22]. Based on these studies, we hypothesized that ankle volume could be used to predict TKA component size because it is a more accurate index than shoe size. Our result confirmed this hypothesis, as ankle size significantly predicted the size of tibial and femoral components in both univariate and multivariate analyses. In another study, BH Naylor et al., by examining 337 TKA candidate patients, showed that the patient’s shoe size had a strong relationship with the size of the TKA implant, which was consistent with the results of our study [10]. JC van Egmond et al. [23] showed a significant positive relationship between the femur and tibia component with shoe size. Similar to the results of our study, they showed that shoe size and ankle volume could be used as a suitable and accurate predictor in preoperative implant sizing for primary TKA.

The present study had limitations. The main limitations of this study were the retrospective design and the small patients’ number. The small number of patients in some subgroups is the other limitation of this study. Also, we did not compare the generated model with the preoperative radiographic templating model. Therefore, we need to determine whether our model better predicts the TKA implant size.

## Conclusion

Demographic characteristics of TKA candidates, including sex, height, weight, and ankle volume, are significantly associated with the size of TKA implants. The model generated using these variables correctly predicts the size of the tibial and femoral components in 77% and 81.1% of cases, respectively. Therefore, these data could predict TKA implant size at a lower cost, time, and difficulty than preoperative radiographic templating.

## Data Availability

The datasets used and/or analysed during the current study are available from the corresponding author on reasonable request.

## Abbreviations

AP: Anteroposterior
BMI: Body Mass Index
ML: Mediolateral
TKA: Total Knee Arthroplasty
